# Projection of seasonal influenza severity from sequence and serological data

**DOI:** 10.1371/currents.RRN1200

**Published:** 2010-12-03

**Authors:** Yuri I Wolf, Anastasia Nikolskaya, Joshua L. Cherry, Cecile Viboud, Eugene Koonin, David J. Lipman

**Affiliations:** ^*^National Center for Biotechnology Information, National Library of Medicine, National Institutes of Health, Bethesda, MD 20894, USA; ^§^Fogarty International Center, National Institutes of Health, Bethesda, MD, USA and ^¶^National Center for Biotechnology Information, National Library of Medicine, National Institutes of Health

## Abstract

Severity of seasonal influenza A epidemics is related to the antigenic novelty of the predominant viral strains circulating each year. Support for a strong correlation between epidemic severity and antigenic drift comes from infectious challenge experiments on vaccinated animals and human volunteers, field studies of vaccine efficacy, prospective studies of subjects with laboratory-confirmed prior infections, and analysis of the connection between drift and severity from surveillance data. We show that, given data on the antigenic and sequence novelty of the hemagglutinin protein of clinical isolates of H3N2 virus from a season along with the corresponding data from prior seasons, we can accurately predict the influenza severity for that season. This model therefore provides a framework for making projections of the severity of the upcoming season using assumptions based on viral isolates collected in the current season. Our results based on two independent data sets from the US and Hong Kong suggest that seasonal severity is largely determined by the novelty of the hemagglutinin protein although other factors, including mutations in other influenza genes, co-circulating pathogens and weather conditions, might also play a role. These results should be helpful for the control of seasonal influenza and have implications for improvement of influenza surveillance.

## Introduction

Kilbourne has described influenza as "...an unvarying disease (three-day fever) - caused by a varying virus" [1]. In 1934, Torrens noted an apparent paradox: although influenza survivors of the epidemic of 1890 largely seemed to escape the 1918 epidemic, annual influenza attacks were common. He proposed that influenza was caused by a "pleomorphic virus" that "... may exhibit mutation from one type to another..." to explain this puzzling pattern of post-influenzal immunity [2]. The challenge this would represent for vaccination became clear by 1947 when a vaccine that was effective in the 1943-1944 and 1945-1946 seasons had no effect for the 1946-1947 influenza season [3]. Salk and Suriano, in a study of the 1946-1947 strain, raised the possibility that "... this strain is a 'mutation' and that this might mean that we will always be immunizing against a disease that occurred the year before" although they remained hopeful that there would be a limited number of antigenic varieties [4].

The variation of influenza by mutation, i.e., antigenic drift, was soon recognized as the basis for the difficulty of predicting epidemics [5], and better understanding the quantitative impact of drift on seasonal epidemics has become an important goal in influenza research. Given that varying levels of cross-protection by earlier viruses are likely to be an important factor in epidemic severity, one experimental strategy has been to immunize subjects with known strains and determine susceptibility to a challenge with another known strain. McLaren et al. [6] vaccinated ferrets with A/Hong Kong/1/68 or A/England/42/72, later challenged with a A/England/42/72-like virus, and indeed found greater protection by the matching strain. A similar, more extensive study has been performed on horses using equine influenza virus in order to estimate the parameters of an epidemiological model of outbreak risk as a function of immune escape [7]. In a study with human volunteers immunized with one of four influenza strains isolated in 1968, 1972, 1973, or 1974, and then challenged with a 1974 strain, Potter et al. [8] found that susceptibility increased linearly with the number of years separating the challenge and vaccine strains. Consistent results were obtained in a similar study by Larson et al. [9].

Field studies of vaccine efficacy have confirmed the results of these experimental approaches (c.f. [10]). As expected, efficacy is substantially higher when the vaccine strain matches the dominant seasonal strain than when there is a measurable mismatch between the vaccine strain and the dominant strain [11]. Gupta et al. [12] compiled an extensive set of vaccine efficacy data and found a strong correlation (R^2^ = 0.81) between vaccine efficacy in a given season and the fraction of amino acid replacements in the antigenic regions of the hemagglutinin protein between the vaccine strain and the dominant circulating strain. Evidence of a more direct quantitative connection between the antigenic novelty of the viruses from a given season and susceptibility to influenza can be found in a prospective study of the 1976 seasonal epidemic by Gill and Murphy [13]. The researchers determined the most recent year of laboratory confirmed influenza for a set of volunteers and found a strong linear relationship between the probability of infection in the 1976 season and the number of years since the previous infection. The above studies suggest that the number of susceptible individuals for a given season, and hence the number of influenza cases, will be strongly correlated with measures of the antigenic distance between prevailing viruses for that season and viruses that circulated in previous seasons. 

In light of the compelling evidence for a quantitative relationship between antigenic drift and seasonal severity, it is perhaps surprising that until recently no study demonstrated a positive correlation between drift and severity using data derived directly from influenza surveillance. A recent study by Wu et al. [14] focuses on  influenza H1N1 and considers major "antigenic strains" based on hemagglutination inhibition (HI) assays -- a method traditionally used by the Centers for Disease Control (CDC) to characterize seasonal viruses for vaccine selection and surveillance purposes. Wu et al. then estimate the excess mortality attributable to each antigenic strain using the fraction of isolates positive for that strain among all those tested in seasons in which the strain was detected. This analysis has shown a strong correlation between estimates of the multi-season excess mortality due to an antigenic strain and its antigenic distance from previous strains for H1N1, and a weaker correlation for H3N2. Given that in recent years, most of the inter-pandemic influenza morbidity was caused by H3N2 viruses [15], we were interested in investigating the relationships between sequence and antigenic variation in the H3N2 HA and the seasonal severity (health burden) of influenza caused by this subtype. In this analysis, we aimed at reaching beyond correlations and developing a practical model for projecting the health burden of influenza H3N2.

Extending previous work on the relationship of antigenic drift and seasonal severity, we here use sequences of hemagglutinin proteins from H3N2 clinical isolates and the corresponding HI data from two countries to demonstrate that one can *reconstruct *with surprising accuracy the severity of a given season. That is, given only data on the antigenic novelty of the viruses from a season we can predict its epidemiological severity.  Furthermore, our statistical model provides a framework for making projections of the severity of the upcoming season using assumptions based on viral isolates collected in the current season.  These results are expected to be helpful in the planning process for the control of seasonal influenza and have implications for influenza surveillance.

## Results

### Epidemiological indices

The seasonal impact of influenza on a population is notably difficult to measure precisely due to the non-specificity of symptoms and the lack of diagnostic tests conducted in routine, and morbidity data are especially scarce. To overcome this problem, we used several epidemiological indicators and validated our predictive algorithm by considering two separate countries: the US and Hong Kong. In the US analysis, we used seven independently-collected epidemiological indicators, each representing a slightly different aspect of influenza A/H3 seasonal disease burden. These indicators were available for 16 consecutive seasons from 1993-1994 to 2008-2009 and comprised:

1. a seasonal indicator of the dominance of influenza A/H3 over the A/H1 subtype for each season (***F-H3***);
2. the seasonal fraction of positive influenza specimens over all respiratory samples tested (***F-Pos***);
3. a proxy for the seasonal number of H3 cases, H3 severity (***S-H3***);
4. influenza-related seasonal excess mortality rate (***R-Mo***);
5. influenza-related seasonal excess hospitalization rate (***R-Ho***),
6. seasonal percent of Influenza-Like Illnesses (***F-ILI***);
7. index of speed of spread (***I-Sp***).

Percent of Influenza-Like Illnesses (*F-ILI*) was calculated as percent of consultations for influenza-like illness out of the total number of consultations reported to the surveillance system for a given season. Fraction of positive specimens (*F-Pos*) was calculated as percent of specimens positive for influenza virus of all specimens collected during the season. (Data source: CDC [16]). An indicator of the dominance of influenza A/H3 over the A/H1 (*F-H3) *was calculated as the logarithm of the ratio of H1 to H3 isolates (Data source: CDC [16]).

H3 epidemic severity (*S-H3*) was calculated as the product of total influenza epidemic severity and the fraction of H3 isolates among all influenza isolates [16]. Total epidemic severity was calculated using two sources of data because no available data source covers the entire period of 1993-2008. For the period of 1997/98 – 2008/09, total epidemic severity was calculated as *F-ILI* x *F-Pos*. For the period of 1993/94-1996/97, we first calculated seasonal excess mortality impact (*R-Mo*, excess death rate from pneumonia and influenza per 100,000) as in [17]. Excess mortality is a traditional indicator of influenza disease burden and is estimated from national vital statistics as mortality in excess of an expected seasonal baseline representing the level of mortality in the absence of influenza [17]. Then, the total severity was estimated as a normalized mortality impact, based on the correlation between mortality impact and the ILI-based severity measure described above,* *over the years where both were available (1997/98-2005/06) (using the geometrical mean of the ratio ILI-based severity/mortality impact for 1997/98-2005/06).

The index of speed of spread (*I-Sp*) was calculated as the standard deviation of the dates of peak pneumonia and influenza mortality rates across 49 continental states (48 states+ DC) [18]. This index has been shown to vary with the intensity of influenza epidemics, with larger epidemics spreading faster across the continental US and resulting in a lower index of spread (lower standard deviation). 

Hospitalization excess rate (*R-Ho*) was derived from the State Inpatient databases maintained by the Agency for Healthcare Research and Quality. We chose 9 states which contributed data for 1989-2008 and represented 30% of the US population and compiled weekly number of admissions with any mention of pneumonia and influenza in the list of diagnoses. We applied the same method used to estimate excess mortality to hospitalization data and derived estimates of seasonal excess hospitalization rates attributable to influenza in the US.

### Analysis of epidemiological data

Epidemiological data were analyzed using the following protocol. First, visual inspection of scatterplots was performed to ensure the absence of gross deviations from linear relationships between pairs of variables. All data vectors were standardized to an average of 0 and a standard deviation of 1; missing data points were assigned the value of 0. Principal Component Analysis (PCA) was performed without further scaling, so variables with more missing data contributed less to the principal components due to reduced variance.

Most of the seven indicators are highly correlated with each other (e.g. Spearman rank correlation *r*_s_[*S‑H3*,*F‑H3*] = ‑0.91 and *r*_s_[*R‑Mo*,*R‑Ho*] = 0.71). We used PCA to transform these correlated variables to a space of uncorrelated variables. The first principal component (*PC1*) accounted for 66% of the total variance; all original indicators are correlated with *PC1* with |*r*_s_| = 0.57 .. 0.94. This variable was used as a single combined epidemiological index (Figure 1) for subsequent analyses with genetic and antigenic information.

**Figure:**
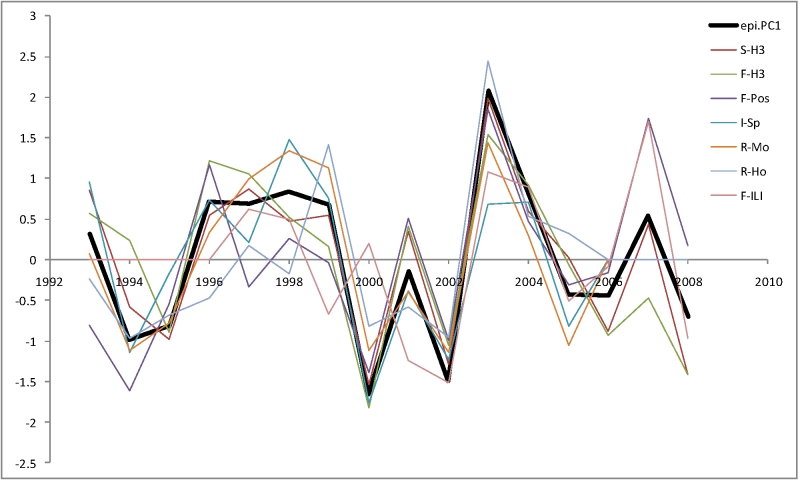
**Figure 1.** First Principal Component (epi.PC1) of seven measures of epidemiological severity

### Genetic distances

HA1 sequences of the H3N2 influenza A virus available from the NCBI Influenza Virus Resource [19] as of June 2009 were used for analysis (see supplementary files [20] [21] [22]). All coding region (CDS) sequences of human H3N2 isolates (excluding short fragments) were downloaded and aligned using the MUSCLE multiple alignment program [23] with subsequent manual editing of the alignment. Aligned HA1 sequences of H3N2 influenza isolates from the US were sorted by seasons (from 1992-1993 to 2008-2009). The Northern Hemisphere influenza season was considered to start in August and end in July of the next calendar year. The distance between two seasons was taken to be the mean number of differences between sequences from one season and those of the other season. Such distances were calculated for all pairs of seasons separated by three years or less.  This calculation included the comparison of every season to itself, which yields a measure of the within-season diversity. Overall amino acid distances (*sa*), amino acid distances in (*se*) and outside (*sn*) of the commonly used set of presumed antigenic epitope sites [24] [25] and nucleotide synonymous (*ss*) distances were computed. For each given season (*n*-th), seven intra- and inter-season distances were calculated (Figure 2).

**Figure:**
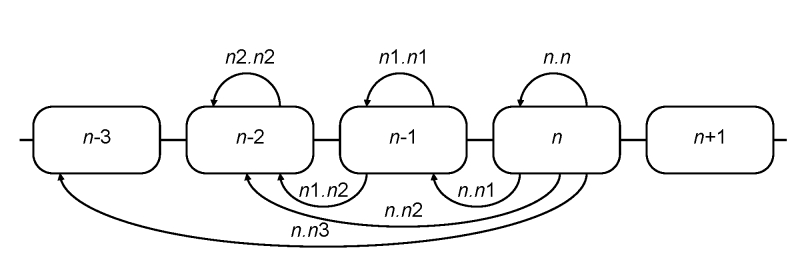
**Figure 2.** Computation of inter-season genetic and serological distances

In combination with the above four types of distances, 28 relative season distance variables were available for each season (e.g. for the 2005-2006 influenza season *se.n*1.*n*1 refers to the average epitope sequence differences in the season of 2004-2005 and *ss.n*.*n*2 is the average synonymous distance between all pairs of isolates from 2005-2006 and 2003-2004 seasons).

### Serological distances

We compiled hemagglutinin inhibition data from a variety of literature sources, including published articles, MMWR summaries, and FDA reports (see supplementary file for references [26]).  A two-dimensional antigenic map was calculated from hemagglutinin inhibition data according to [27]. From this, antigenic distances were calculated for all pairs of isolates. Mean intra- and inter-season pairwise distances were then calculated for North America (*aa*) and Northern Hemisphere (*an*) isolates, corresponding to all of the  types of distances calculated for sequence data (Figure 2) except that the *n*.*n*3 distances were omitted. In combination with the above two sources of the isolates, 12 relative season distance variables were available for each season.

### Model selection

Overall, we had 40 explanatory variables, comprising 7*4=28 genetic variables and 6*2=12 serological distance variables, and only 16 epidemiological observations. Due to the potential for severe overfitting, the standard approach of stepwise reduction of a model containing all variables was not feasible. Instead we adopted a two-stage approach to find the optimal balance between the goodness of fit and the number of degrees of freedom in the model. At the first stage we used a transformation of the original distance variables to determine which of them contributed most to the prediction of the epidemiological variable; at the second stage we applied this information to select the most statistically robust predictors using the original variables. 

## Transformation of distance variables

For each relative season, combination the data from different variables (*sa*, *se*, *sn* and *ss* for genetic and *aa* and *an* for serological data) are strongly correlated (e.g. *r*_s_[*sa.n*.*n*1,*ss.n*.*n*1] = 0.74). Thus each of the 28 genetic variables and 12 serological distance variables were subject to PCA (Figure 3). The first 2 principal components were retained for each data series, reducing the number of potential explanatory variables from 40 to 26.

**Figure:**
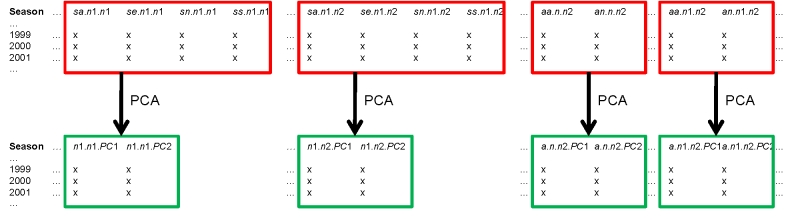
**Figure 3.** Principal Component Analysis (PCA) of genetic and serological distance variables

##  Retrospective model: first stage

We tested linear models containing all combinations of 3 (2600 models), 4 (14950 models), 5 (65780 models) and 6 (230230 models) variables. Model fitting was performed on the R platform using the *lm*() function. Within each class the models were ranked by decreasing residual sum of squares. Additionally, 19 variables most frequently present among the top 50 6-variable models were used to generate all combinations of 7 (50388 models) and 8 (75582 models) variables; 7- and 8-variable models were ranked in the same manner.

To test the models statistically, we used two variants of a jackknife resampling procedure [28]. The first variant tests the statistical robustness of the relationships between the explanatory variables and the target variable in general. The second tests the statistical robustness of a model using a particular set of explanatory variables.

In the first variant we removed data for each single season from our dataset one-by-one and used the remaining 15 seasons to find the best-fitting model (both the set of the explanatory variables and coefficients). Then we used this model to predict the epidemiology for the remaining season (leave-one-out jackknife scheme). The sum of squares of the deviations of these predictions from the observations was accumulated over all 16 seasons to serve as the robustness indicator.

In the second variant we generated all possible bipartitions of the 16 epidemiological observations into two classes of equal size. With each bipartition, one half of the seasons data was used to train the model using the fixed set of the explanatory variables; then these model coefficients were used to predict the epidemiology for the remaining 8 seasons (leave-half-out jackknife scheme). The residual sums of squares for the control half of the data were accumulated over all bipartitions to serve as the indicator of the model robustness. We tested the top 10 models from each class and ranked them by decreasing total residual sum of squares.

In the leave-one-out jackknife test the 5-variable model family demonstrates a highly robust behavior, accounting for 0.96 of the of the variance in *epi.PC*1. The following 5-variable model demonstrated the best performance among all tested models:

*epi.PC*1’ ~ *n.n*2.*PC*1 + *n.n*2.*PC*2 + *n*1*.n*1.*PC*1 + *a.n*1*.n*1.*PC*1 + *a.n*1*.n*1.*PC*2 + 0

Hence this model includes genetic distance between the current season and 2 seasons before, and intra-season genetic and serological distances between isolates circulating in the previous season in the US (genetic) or North America and Northern Hemisphere (serological). When trained on the full complement of 16 seasons (Figure 4), this model explains 0.98 of the original variance (F-statistics p-value of 3x10^-9^), the coefficients being -1.36, -1.54, -0.32, -1.28 and -1.69 respectively (PCA gives arbitrary signs to the principal components, so the signs of coefficients do not indicate the kind of relationship by themselves). In the leave-half-out jackknife test it explains, on average, 0.89 of the variance in *epi.PC*1.

**Figure:**
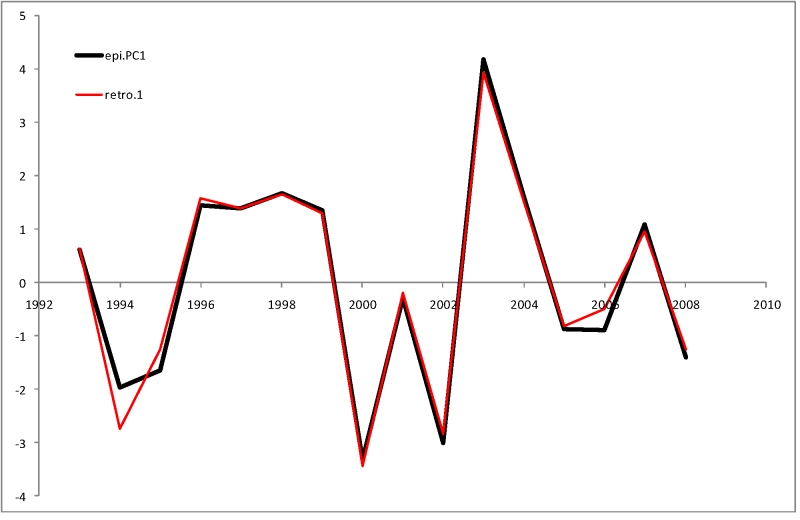
**Figure 4.** Comparison of first stage retrospective model (retro.1) with observed epidemiological severity (epi.PC1)

To further ascertain the statistical validity of the relationship between the genetic and serological data and epidemiological severity we implemented an additional test procedure. The epidemiological data vector (*epi.PC*1) was permuted and all combinations of 5 out of 26 explanatory variables were tested as potential reconstruction models for the scrambled *epi.PC*1 data. Only 36 out of 100,000 permutations yielded a fit as good as that obtained with the unpermuted data.

The above shows that the sequence and antigenic data explain part of the variance in morbidity among seasons.  We can reject the hypothesis that the true *R* ^2^ equals zero with high statistical confidence, and we estimate that the true *R* ^2^ is 0.96. In order to obtain a statistical lower bound on the true *R* ^2^, we generated epidemiological severity values that mimicked various levels of unexplained variance, and determined the lowest *R* ^2 ^value that could not be rejected at the 5% level by the *R* ^2^ observed for the real data (i.e., the lowest value for which the *R* ^2^ of the fit was as good as the observed 98% in at least 2.5% of the replicates).  To the epidemiological values predicted by the fitted model, which can be fit with *R* ^2^ = 1, we added independent pseudo-random numbers.  Two distributions for these were used: a normal distribution and a Laplace (double exponential) distribution.  The Laplace distribution has an excess kurtosis of 3.0, and is used because the observed residuals have excess kurtosis (approximately 2.5).  For each trial quantity of added noise, many replicates were generated, and each was independently fit to the sequence and antigenic data, with no constraint on the choice of combinations of explanatory variables.

The 95%-confidence lower bounds for *R* ^2^ thus obtained were 0.88 with normally-distributed deviations and 0.84 with Laplace-distributed deviations.  Upper bounds, obtained similarly, were both close to 0.99.  We conclude that the fitted model likely accounts for the vast majority of the season-to-season variance in epidemic severity.

## Retrospective model: second stage

In the first stage of the analysis, we found that genetic distances between the current season and a season two years ago (*n.n*2) and the genetic and antigenic diversity one season ago (*n*1*.n*1) yield the most statistically robust retrospective reconstruction of H3N2 influenza epidemiology. In the second stage, we apply a similar approach to prediction of influenza epidemiology in the next season.

In the second stage, we apply the standard stepwise model reduction approach to find the optimal set of original variables. We started with a model containing 8 variables based on the best-fit model identified in the 1st stage (*se.n.n*2, *sn.n.n*2, *ss.n.n*2,* se.n*1*.n*1,* sn.n*1*.n*1,* ss.n*1*.n*1,* aa.n*1*.n*1 and *an.n*1*.n*1) and removed one by one the variables that contribute the least to the model (i.e. with the lowest absolute *t*-value of the coefficient). Each derived model was compared to its parent using the *anova*() function of R; if the reduced model was not rejected at significance threshold of 0.05, the process continued. The final model that cannot be reduced any further without a significant drop of the goodness of fit contains the following 5 variables: 

**

*epi.PC*1’ ~ *se.n.n*2 + *sn.n.n*2 + *ss.n.n*2 + *se.n*1*.n*1 + *aa.n*1*.n*1 + 0

When trained on all 16 seasons (Figure 5), this model explains 0.98 of the original variance (F-statistics p-value of 3x10^-9^), the coefficients being 1.26, 1.78, 0.81, -0.41 and 2.08, respectively. In the leave-half-out jackknife test the model explains, on average, 0.91 of the variance in the remaining 8 seasons.

**Figure:**
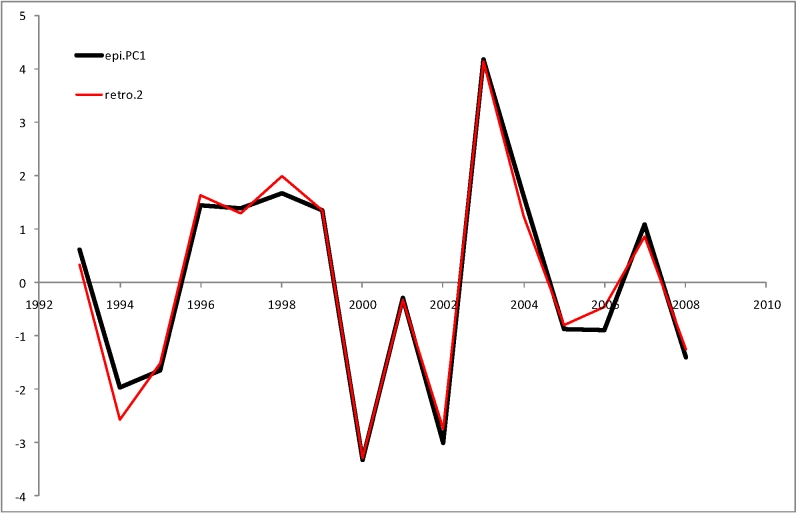
**Figure 5.** Comparison of second stage retrospective model (retro.2) with observed epidemiological severity (epi.PC1)

## Independent test: Hong Kong data

To test whether these results pertain only to US or are more generally applicable to H3N2 influenza epidemiology, we collected a similar data set from Hong Kong and mainland China. Three epidemiological indicators (*S-H3*, *F-H3* and *F-Pos*) were obtained for 13 seasons (from 1997 to 2009) using data from Hong Kong Centre for Health Protection [29].  Influenza epidemics in Hong Kong usually show two peaks (February-March and May-June). Both peaks were combined into the same season, and therefore a season corresponds to a calendar year in this analysis. 

Because Hong Kong Centre for Health Protection data lacks ILI raw numbers, only %ILI for each month is available. Therefore, we calculated the total epidemic severity (%ILI*%Positive Specimens) for each month, and then totaled these numbers for a year to obtain. (As a test to show that this method gives results similar to the regular method (using cumulative raw numbers for each season to calculate %ILI and %Positive Specimens), we checked  the correlation between the epidemic severity numbers calculated using the two methods for the USA data. The correlation between the two methods is very high (r=0.97). The H3 epidemic severity (*S-H3*) was calculated as the total influenza epidemic severity prorated by the fraction of H3 isolates among all isolates. These data were converted to a single epidemiological index using PCA (Figure 6). Interestingly, despite the fact that Hong Kong, like USA, is a non-tropical Northern Hemisphere country, the epidemic severity measures between the two are relatively weakly correlated (*r_s_*[*epi.PC*1*.USA*,*epi.PC*1*.HK*] = 0.36 with the p-value of 0.21). Thus the Hong Kong dataset provides a largely independent test of the model applicability.

Sequence and antigenic distances were computed in a manner similar to that for the US data (the list of Hong Kong isolates used for genetic distance calculation is in the supplementary file [21]). Again, in this analysis, a season corresponds to a calendar year. Due to a low number of serologically tested isolates from Hong Kong proper, antigenic data were computed for a combined set of Hong Kong and mainland China isolates. The seasonal epidemiology seems to be the same in Hong Kong and Southern mainland China, and only Northern mainland China follows the standard Northern Hemisphere pattern [30]. Moreover, the Northern China epidemics follow the Southern China epidemics and not vice versa [30]. Based on this observation, two possible ways of merging these data sets were implemented, producing two versions of the serological distances (*ac* and *as* in contrast to *aa* and *an* serological distances for US). The first one (*ac*) corresponds to the scenario where for all isolates (Hong Kong and China) the season was considered to correspond to a calendar year, following the Hong Kong pattern. The second one (*as*) considers Hong Kong isolates following the Hong Kong pattern and China isolates following the Northern Hemisphere pattern with seasons starting in the fall.

**Figure:**
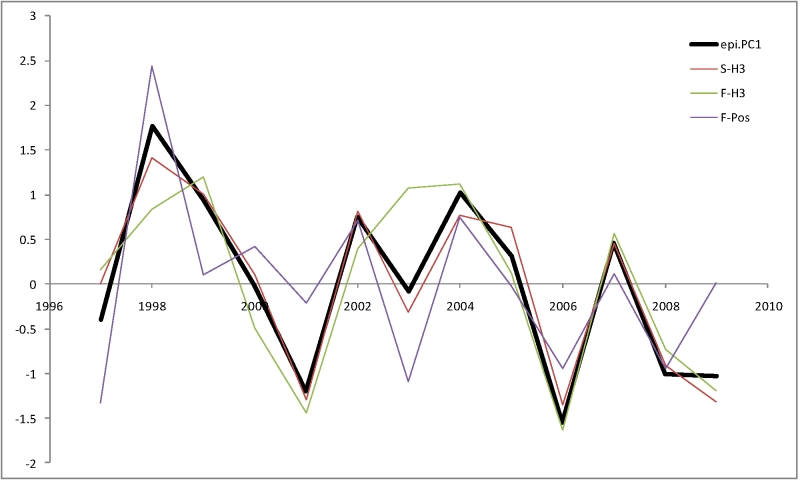
**Figure 6.** First Principal Component (epi.PC1) of three measures of epidemiological severity for Hong Kong

There are several possible ways to apply the model derived from the US data to the Hong Kong data. One would be to directly compute the model prediction using the formula:

*epi.PC*1' = 1.26*se.n.n*2 + 1.78*sn.n.n*2 + 0.81*ss.n.n*2 - 0.41*se.n*1*.n*1 + 2.08*aa.n*1*.n*1

and replacing *aa.n*1*.n*1 (US data) with either *ac.n*1*.n*1 or *as.n*1*.n*1. Using *as.n*1*.n*1 for the serological distance variable, this formula produces a prediction that is reasonably well correlated with the real epidemiology data (*r*_s_[*epi.PC*1,*epi.PC*1’] = 0.60, with the p-value of 0.012 in a permutation test; Figure 7, "retro.direct" plot). This model allows rough prediction of the ups and downs of the H3N2 influenza epidemics in 1997-2009, but gives a relatively poor quantitative estimate.

When the model is allowed to use the actual epidemiological data from Hong Kong to adjust its coefficients (yielding 1.50, 1.46, -0.82, 0.34 and 0.64 respectively), the prediction is improved (Figure 7, "retro.adjust" plot). The adjusted model explains 0.75 of the original variance with F-statistics p-value of 2x10^-2^.

Finally, the stepwise reduction of the full model containing the genetic and serological distances from the corresponding seasons (*se.n.n*2, *sn.n.n*2, *ss.n.n*2,* se.n*1*.n*1,* sn.n*1*.n*1,* ss.n*1*.n*1,* ac.n*1*.n*1 and *as.n*1*.n*1) leads to the following 4-parameter model (Figure 7, "retro.stepwise" plot):**

*epi.PC*1’ ~ *se.n.n*2 + *sn.n.n*2 + *ac.n*1*.n*1 + *as.n*1*.n*1 + 0

The model coefficients are 1.25, 0.75, 0.76 and 1.10 respectively; it explains 0.78 of the original variance with F-statistics p-value of 5x10-3. When trained on 8 out of the 13 seasons (leave-5-out jackknife test scheme) , this model explains 0.42 of the original variance on average.

**Figure:**
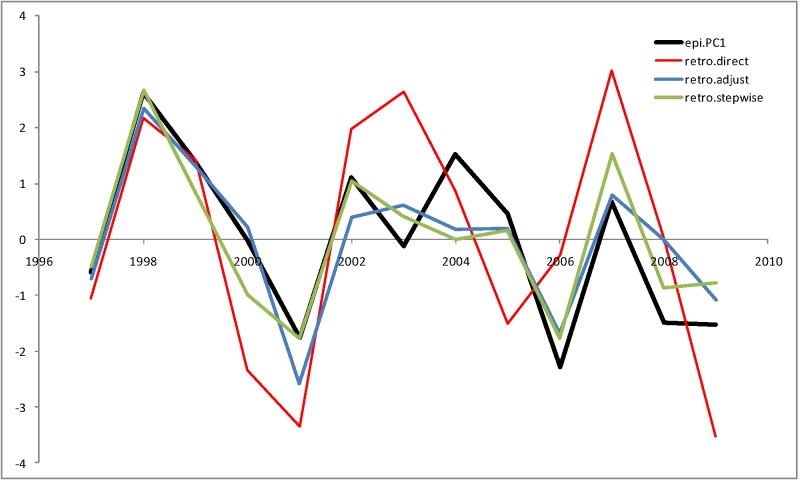
**Figure 7.** Comparison of different retrospective models with observed Hong Kong epidemiological severity (epi.PC1)

## Projection model: Southern Hemisphere isolates

The retrospective model allows us to accurately reconstruct the epidemiological severity of a season using sequence and serological data from that season's viral isolates.  This provides a framework to project severity for the upcoming season based on assumptions about the population of viruses that might circulate in that season. An obvious assumption to assess would be that the population of viruses in an upcoming Northern Hemisphere season is identical to the population of viruses seen in the preceding Southern Hemisphere influenza season.  To this end we use the sequence and serological data for the isolates observed in the Southern Hemisphere in the season preceding the flu season in the Northern Hemisphere. By September-October the Southern Hemisphere isolates are, at least in principle, available for analysis before the start of the Northern Hemisphere flu season. In any given season these Southern Hemisphere isolates are used as a substitute for the current Northern Hemisphere isolates as shown in Figure 8.

**Figure:**
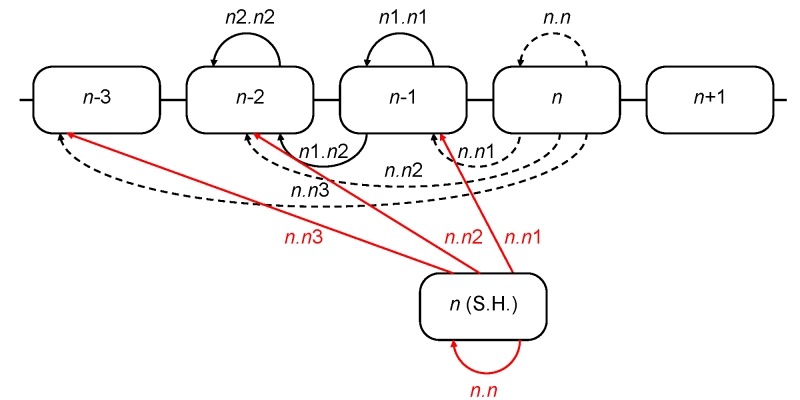
**Figure 8.** Computation of inter-season genetic and serological distances using the isolates from the Southern Hemisphere. Distances computed along the red arrows replace those, computed along the dashed arrows

For the purpose of this analysis, we considered Southern Hemisphere as comprising only non-tropical Southern countries because of a different influenza epidemic pattern in the Tropics [31]. Southern Hemisphere seasons were considered to be equivalent to calendar years, and correspond to the subsequent Northern Hemisphere seasons (e.g., 2003 Southern Hemisphere season corresponds to the 2003/04 Northern Hemisphere season). There were no Southern Hemisphere sequences from 2008, and we used the only January 2009 sequence as a substitute for 2008 because January is borderline between the two Southern seasons (2008 and 2009). List of Southern Hemisphere isolates used for genetic distance calculation is in the supplementary file [22]. Genetic and antigenic distances were computed as before.

We apply the second stage retrospective model (list of explanatory variables together with the coefficients) trained on the original dataset to this modified dataset to obtain the model projections to the current season using the Southern Hemisphere data from the preceding season (Figure 9).

**Figure:**
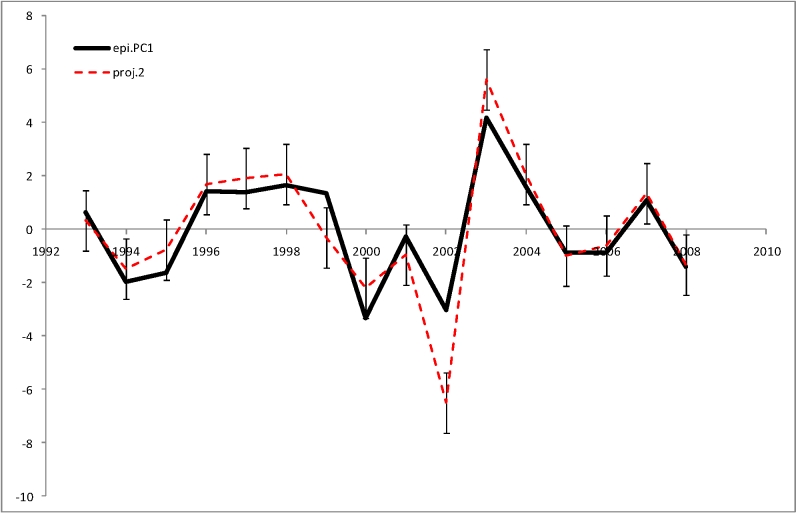
**Figure 9.** Comparison of second stage projection model (proj.2) with observed epidemiological severity (epi.PC1). Error bars show the root mean square deviation of the projections from the real observations

The projections explain 0.66 of the original *epi.PC*1 variance and shows an excellent correlation with the epidemiological observations (*r*_s_[*epi.PC*1,*epi.PC*1’] = 0.95, with the p-value <0.0001 in a permutation test).

## Projection using previous seasons.

Although projections based on Southern Hemisphere data correlate quite well with the actual epidemiological observations in the Northern Hemisphere, it would be useful to be able to make these projections earlier if possible, i.e. by the time when the decisions concerning the next season vaccines are made.  A straightforward application of our approach (i.e. using only the data from preceding years to predict the morbidity for the upcoming year) proved to be insufficiently robust statistically (not shown). Often, however, multiple lineages co-circulate in a given season, and  the epidemiological picture may be different depending on which lineage dominates the following season. Which of the co-circulating clades (if any) will become dominant in the next season is not known in advance; however one can construct explicit hypotheses about the upcoming season by assuming each of these clades taking over. Thus, it is possible to employ our model to explore the spectrum of possible predictions based on such assumptions.

As a demonstration of this approach, we divided the USA isolates from the 2002-2003 season into two subgroups - 9 Panama-like (members of the larger Sydney-like class) (supplementary file [32]) and 7 Fujian-like viruses (supplementary file [33]), based on the reconstructed phylogenetic tree for HA1 (A/H3N2 influenza) (supplementary file [34]) as described in [35].  Each of the subgroups was used to substitute for the real 2003-2004 season isolates in our data. The raw sequence distances computed for these subgroups were normalized using means and standard deviations previously determined for the full dataset. Then we used the list of variables and the coefficients determined for the second stage retrospective model to predict the severity of the 2003-2004 epidemics. The two subsets correspond to the two epidemiological hypotheses: one assumes that the 2003-2004 flu season will be dominated by Panama-like H3N2 virus, whereas the other assumes the Fujian-like virus prevalence. The results of these projections are shown on Figure 10.

**Figure:**
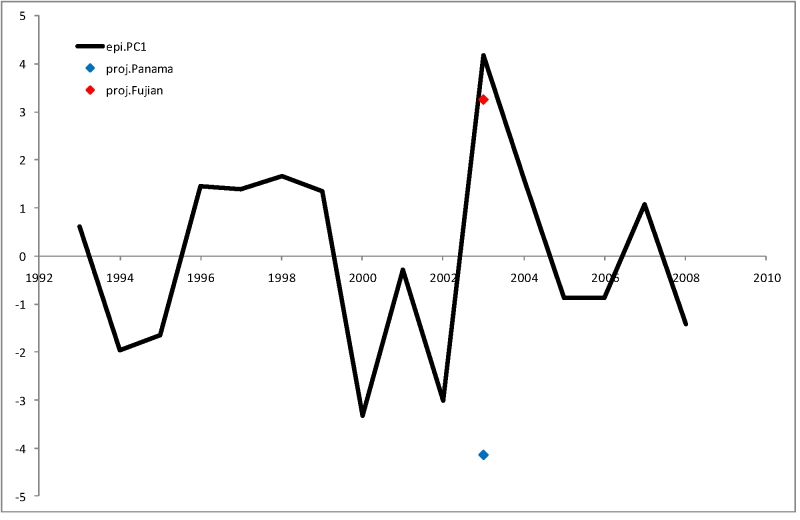
**Figure 10.** Comparison of the previous-season projection models (proj.Panama and proj.Fujian) with observed epidemiological severity (epi.PC1).

Using the Panama projection predicts a very mild flu season for 2003-2004; in contrast, the Fujian projection predicts a severe epidemics. In reality the 2003-2004 flu season was dominated by the Fujian-like isolates worldwide and was the strongest on record for our range of dates in the USA.

All data series used in this work may be found in supplementary file [36].

## Discussion

As reviewed above, an extensive body of research supports the notion that seasonal severity of Influenza A is strongly correlated with the degree of antigenic drift.  Nevertheless, it is surprising that even applying a highly conservative approach (i.e. leave-half-out jackknife test), over 90% of the variance in epidemic severity can be explained by the antigenic and genetic novelty of the hemagglutinin protein. This implies that factors such as climate, school cycles, other co-circulating pathogens, and changes in influenza genes other than the hemagglutinin gene all have at best minor effects on seasonal morbidity, although they may affect epidemic timing.  A number of studies have shown that temperature and humidity play important roles in the spread of influenza (cf. [37] [38] [39] [40]), however, for the scale of our analysis (e.g. geographically across all of North America and temporally for entire seasons), it might be difficult to detect a significant impact.   Other co-circulating pathogens might reduce the number of seasonal influenza cases, e.g. owing to a direct impact on the host immune system or because of indirect effects on the host as the "vector" for spread. This "interference" effect has been reported for respiratory syncytial virus and rhinoviruses [41] [42] [43] [44] but, given our results, this interference does not appear to have been significant during the seasons covered in this study.

Competition between Influenza A virus subtypes has been noted in several focused studies [45] [46] [47] as well as in a broader analysis of surveillance data [48]. However, the impact of competition from H1N1 on variation of variation of H3N2 seasonal severity is unlikely to have been significant during the time interval studied here because H3N2 was dominant most seasons, and furthermore, there is little evidence of positive selection for H1N1 antigenic novelty [49] [35]. In addition, Wolf et al.[35] have shown that during this period, H1N1 tended to dominate only after mild H3N2 seasons, so the competition appeared to be one-sided.

Although our results show that the degree of hemagglutinin novelty explains most of seasonal morbidity, other viral proteins have been shown to play important roles.  For example, consider the evolutionary history of the Fujian H3N2 strain:  initially an antigenically novel minor variant, succeeded by a reassortant containing the novel Fujian HA gene, which in turn is succeeded by a later "pure" Fujian variant in which all genes are derived from the original Fujian strain.  An analysis of these events led to a proposal that deleterious mutations in genes other than HA prevented the earliest members of this clade from dominating despite their antigenic novelty but subsequent compensatory mutations ultimately led to its dominance over the reassortant strain [35].  Recent experiments on the later Fujian variant support the idea that mutations in the polymerase PA gene were responsible for the decreased fitness of the original Fujian strain [50].   

Antigenic diversity in the preceding season is an important component of our statistical model which is consistent with the above observation that an antigenically novel strain may circulate for a season as a minor variant before compensating mutations allow it to dominate the following season.  Mutations in the hemagglutinin gene itself might have effects on fitness beyond that of changing antigenicity.  For example, mutations have been identified in the HA gene that might allow the virus to evade the host's immune response but also modify binding to the host receptor leading to decreased replication fitness (cf. [51] [52] [53]).  Our results suggest that this could be a common feature of early drift variants.

Antigenic diversity in the preceding season is not the only warning of a rise in morbidity but it is difficult to extract other clear signals or rules from our analysis.  In addition to the underlying complexity of the system, the sequence and HI data we have for clinical isolates are not sufficiently comprehensive or representative.  For example, in the 2002-2003 season, according to the CDC's antigenic characterization of the 2002 - 03 U.S. Influenza season [16], 85% of the H3N2 clinical isolates were similar to A/Panama/2007/99 and 15% were similar to A/Fujian/411/2002. However, our sequence data were about half Panama and half Fujian whereas virtually all of the HI data we were able to obtain were similar to Fujian. 

While more representative input data could improve the performance of our statistical model, refinements in other aspects of the method may also be useful.  For example, other sequence-derived measures of novelty might work better than our simple amino acid replacements-based approach.  The method of Gupta et al. [12] yielded equivalent results but there are additional sequence-based methods that should be evaluated as well (cf.[54]). 

Because our method provides an accurate reconstruction of epidemiological severity given sequence and serological data, one can evaluate the epidemiological consequences of various assumptions about the population of viruses for the upcoming season.  As seen in Figure 9, projections using data from Southern Hemisphere isolates provide realistic estimates of severity for the Northern Hemisphere.  When there is significant diversity among co-circulating clades in a given season, one can assess different scenarios for the upcoming season, as was done using data from 2002-2003 seasonal isolates to project severity for the 2003-2004 season.  The panel advising the US Food and Drug Administration on vaccines had difficulties that season because the Fujian strain - a minor strain in 2002-2003 - was antigenically novel but was difficult to grow in chicken eggs.   Scientists at the CDC influenza branch had been able to passage the Fujian strain through dog kidney cells and obtain egg-adapted virus but concern about contamination steered the FDA advisory committee towards a decision to use the Panama strain ( cf [55]).  The ability to make accurate projections of epidemiological severity might have been helpful to further inform this decision.

Although, in some cases, projections based on co-circulating clades could be helpful prior to the availability of Southern Hemisphere isolates, thus assisting in vaccine strain decisions, in other situations, such as the 1997-1998 Sydney season, the rapid emergence of a new dominant strain makes earlier projections effectively useless.  Deeper sampling of representative viral isolates might provide an earlier warning of such novel clades, and more sophisticated models using additional data might also eventually prove helpful. 

Finally, we note that our predictions of influenza morbidity based on sequence and serological data are limited to the inter-pandemic period, and that our model cannot be used to project the severity of an emerging pandemic influenza virus, such as 2009 H1N1pdm. In addition, it remains unclear whether this model rooted in empirical data collected before the 2009 pandemic will be suitable to predict the severity of influenza A/H3N2 season in the post-pandemic period. Both H3N2 and H1N1 have begun to co-circulate in 2010 [56], and further studies will need to determine whether the dynamics of these two subtypes has changed and whether the algorithms proposed here need to be revised to accommodate these changes.

The results presented here should be useful in the planning process for seasonal influenza.  This work, along with the extensive earlier research that revealed the correlation between antigenic drift and seasonal severity, provides an objective function for refining measures to characterize antigenicity and seasonal morbidity.  Given that we can now use surveillance data to make useful projections of influenza morbidity, we should begin to consider those changes to surveillance that maximize the effectiveness of this approach.

## Acknowledgements

We thank Dr. A.I. Klimov (Influenza Division, Centers for Disease Control and Prevention) for kindly providing the haemagglutination-inhibition assay data of Influenza A(H3N2) isolates performed in 1998 and 1999 at CDC. We thank Dr. Cheong-Chi Lau and Dr. Flemy Yip (Hong Kong Centre for Health Protection) for assistance with Hong Kong influenza surveillance data. We thank Dr. Derek Smith (University of Cambridge, UK) for advice on the antigenic distance calculation and for kindly providing haemagglutination-inhibition assay data used in [27]. We thank Dr. John Spouge (National Center for Biotechnology Information, NLM, NIH) for helpful discussions of statistical analysis procedures. 

## Funding information

This research was supported by the Intramural Research Program of the NCBI/NLM/NIH and FIC/NIH.

## Competing interests

 David Lipman, corresponding author, is one of the Editors of *PLoS Currents: Influenza*.
